# Effect of Bupivacaine-Soaked Polypropylene Mesh on Postoperative Pain in Patients Undergoing Lichtenstein Repair for Inguinal Hernia

**DOI:** 10.7759/cureus.93750

**Published:** 2025-10-03

**Authors:** Prashant Kumar, Rohit Srivastava, Neelabh Agrawal, Sanjay K Bhatt, Priyanka Rai, Sunil Singh

**Affiliations:** 1 General Surgery, Dr. Ram Manohar Lohia Institute of Medical Sciences, Lucknow, IND

**Keywords:** bupivacaine-soaked mesh, inguinal hernia surgery, lichtenstein hernia repair, postoperative pain, visual analogue scale (vas)

## Abstract

Background: Inguinal hernia repair is among the most common general surgical procedures worldwide, with the Lichtenstein tension-free mesh repair regarded as the gold standard due to its low recurrence rates and favorable outcomes. However, postoperative pain remains a significant concern. The use of bupivacaine-soaked mesh has been proposed as a technique to enhance analgesia and facilitate faster recovery.

Objectives: This study aimed to evaluate the efficacy of 0.5% bupivacaine-soaked polypropylene mesh in reducing postoperative pain and hospital stay in patients undergoing Lichtenstein repair for unilateral inguinal hernia.

Methods: A prospective cohort study was conducted over 18 months in a tertiary care teaching hospital in Northern India, involving 86 adult patients undergoing elective unilateral inguinal hernia repair. Participants were divided into Group A (bupivacaine-soaked mesh) and Group B (non-soaked mesh). Pain was assessed using the Visual Analogue Scale (VAS) at six, 12, and 24 hours postoperatively. Additional analgesic requirements and hospital stay duration were also recorded. Statistical analysis was performed using IBM SPSS Statistics for Windows, version 24.0 (IBM Corp., Armonk, New York, United States).

Results: Both groups were comparable in baseline characteristics. Group A showed significantly lower VAS scores at six hours (3.95 ± 0.53 vs 4.53 ± 0.68), 12 hours (3.56 ± 0.50 vs 4.09 ± 0.8), and 24 hours (1.65 ± 0.48 vs 2.32 ± 0.52) (p < 0.05). Diclofenac requirement was significantly lower in Group A (p = 0.0004), and 67.4% were discharged by day 3 compared to 4.7% in Group B (p < 0.0001).

Conclusion: The use of 0.5% bupivacaine-soaked mesh in Lichtenstein repair significantly reduces postoperative pain and shortens hospital stay, supporting its role as an effective adjunct in inguinal hernia surgery.

## Introduction

Hernias, characterized by the abnormal protrusion of internal organs or tissues through weakened areas of the abdominal wall, represent a prevalent clinical entity in general surgery. In India, approximately 15-20% of the general population is estimated to be affected by hernias, with inguinal hernias being the most common subtype [1]. These occur predominantly in the groin region and are classified as direct or indirect based on their anatomical relation to the inferior epigastric vessels [2]. Inguinal hernias display a marked male predominance, with a lifetime risk of about 27% in men compared to 3% in women [2]. They may present as reducible swellings in the groin, often exacerbated by physical exertion, and can lead to complications such as obstruction or strangulation if not timely managed [3].

Surgical repair remains the cornerstone of inguinal hernia treatment. Among the available techniques, the Lichtenstein tension-free mesh repair has emerged as the gold standard, owing to its simplicity, low recurrence rates, and favorable postoperative outcomes [4,5]. This method involves placing a polypropylene mesh over the inguinal floor without creating tension on the tissues, thereby promoting durable repair and faster recovery. The use of mesh significantly reduces recurrence rates by reinforcing the abdominal wall and withstanding intra-abdominal pressure during physical activity. Most Lichtenstein procedures are performed under local anesthesia, which further enhances early postoperative recovery and reduces hospital stay [6,7].

Despite these advancements, postoperative pain remains a notable concern. Adequate pain control is crucial for early mobilization, reduced hospital stay, and improved patient satisfaction. Various pharmacological modalities, including opioids, nonsteroidal anti-inflammatory drugs (NSAIDs), and local anesthetics, are employed in multimodal analgesia protocols. A promising technique to improve local pain management involves the use of 0.5% bupivacaine-soaked polypropylene mesh, which may offer prolonged analgesic effects and reduce systemic analgesic requirements [8,9]. Bupivacaine is a long-acting amide local anesthetic that blocks voltage-gated sodium channels, inhibiting depolarization and nerve impulse propagation. It provides sensory blockade lasting six to eight hours, with minimal systemic absorption when used for mesh soaking [10,11]. However, evidence supporting its effectiveness remains limited and inconclusive.

Therefore, this study aims to assess whether the use of 0.5% bupivacaine-soaked polypropylene mesh in Lichtenstein repair for unilateral inguinal hernia provides superior postoperative pain relief compared to conventional non-soaked mesh. The study will evaluate pain using the Visual Analogue Scale (VAS), additional analgesic requirements, and postoperative hospital stay, thereby contributing to evidence-based improvements in hernia surgery.

## Materials and methods

Study design and setting

This prospective comparative study with two parallel groups was conducted over a period of 18 months in the Department of General Surgery at a tertiary care teaching hospital, Dr. Ram Manohar Lohia Institute of Medical Sciences, Lucknow, Uttar Pradesh, India. The primary objective was to evaluate the impact of 0.5% bupivacaine-soaked polypropylene mesh on postoperative pain in patients undergoing Lichtenstein repair for unilateral inguinal hernia. Patients were divided into two groups based on the type of mesh used: those receiving bupivacaine-soaked mesh and those receiving standard non-soaked mesh. Postoperative pain was assessed using the Visual Analogue Scale (VAS), along with evaluation of analgesic requirements and duration of hospital stay, to determine the effectiveness of the intervention.

Study population

The study included adult patients aged 18 years and above undergoing elective Lichtenstein repair for unilateral inguinal hernia. Exclusion criteria were patients with irreducible, obstructed, or strangulated hernias, recurrent hernias, those planned for additional surgical procedures in the same sitting, current or regular use of analgesics for other conditions, and known hypersensitivity to bupivacaine. The sample size was calculated using data from a previous study [7], which provided estimates for variability in pain scores. Based on these parameters, a minimum of 43 patients per group was determined to be necessary, resulting in a total sample size of 86 patients. The selected study population represented a typical patient demographic encountered in surgical practice in Northern India, thereby enhancing the relevance and applicability of the study findings.

Sample size formula used was:

$n = \frac{\bigl(Z_{\alpha/2}+Z_{\beta}\bigr)^{2}\,\sigma^{2}}{d^{2}}$, where Z_α/2_ is the critical value of the normal distribution at α/2 (for a confidence level of 95%, α=0.05, and the critical value is 1.96), Z_β_ is the critical value of the normal distribution at β (for power of 80%, β=0.2 and the critical value equals to 0.84), σ is the population variance calculated using standard deviation for mean VAS score among patients who underwent bupivacaine soaked mesh is used and non soaked mesh is used during inguinal hernia repair (value is 1.33), and d is the hypothesized difference the study wants to detect (value is 0.7).

To detect a hypothesized difference of 0.7 between the cases (bupivacaine-soaked mesh) and controls (no soaked mesh) (variance=1.33), significant with a 95% confidence interval (CI) and power of 80%, the required minimum sample size was 43 patients in each group. The total minimum sample size was 86 patients.

Data collection

All eligible patients with unilateral inguinal hernia scheduled for elective Lichtenstein repair were enrolled after obtaining informed written consent. Clinical and radiological evaluation, including high-resolution ultrasonography, was performed. Patients were randomized into two groups using a simple random allocation method. Group A received 0.5% bupivacaine-soaked polypropylene mesh, while Group B received standard non-soaked polypropylene mesh.

For Group A, the mesh was soaked in 10 mL of 0.5% bupivacaine for 10 minutes before placement. The concentration was chosen based on previously published protocols [7], as a higher volume but lower concentration may risk fluid accumulation. The 10-minute soaking time was adapted from earlier studies evaluating local anesthetic mesh impregnation [7], where adequate absorption was demonstrated. Mesh size and fixation technique were standardized for both groups, with a polypropylene mesh of uniform size fixed using interrupted polypropylene sutures.

All procedures were performed under spinal anesthesia with 3 mL of 0.5% hyperbaric bupivacaine administered at the L3-L4 interspace in the sitting position under strict aseptic precautions. Preoperative, intraoperative, and postoperative data were recorded in a structured proforma. Postoperative pain was assessed by a trained resident not involved in surgery or patient care, using the VAS at six, 12, and 24 hours. However, blinding of the assessor was not attempted, as blinding was not feasible due to the visible difference in mesh preparation. This remains a limitation of the study.

All patients received IV paracetamol 1 g every eight hours for the first 24 hours postoperatively. IV diclofenac 75 mg was administered as rescue analgesia if required, with time and frequency noted. Dependent variables included VAS scores, additional analgesic use, and hospital stay duration; independent variables included age, sex, comorbidities, smoking history, and alcohol use.

Data analysis

Data was entered and analysed using IBM SPSS Statistics for Windows, version 24.0 (IBM Corp., Armonk, New York, United States). Descriptive summary using frequencies, percentages, graphs, mean, and standard deviation was used to present study results. Probability (p) was calculated to test statistical significance at the 5% level of significance. Categorical variables were analysed using the chi-square test. Continuous variables were compared between the two groups using an independent t-test. Repeated measures ANOVA was used to assess the effect of the intervention within the groups.

## Results

The age distribution in the present study demonstrated a wide range across both groups. In Group A (bupivacaine-soaked mesh), the majority of participants were in the age group of 51-60 years (27.91%), followed by the age groups of 18-30 years and >60 years (20.93% each), with a mean age of 45.9 ± 15.41 years. In Group B (non-soaked mesh), most participants also fell within the 51-60 years age group (30.23%), followed by those aged 31-40 years (18.6%), with a mean age of 46.3 ± 12.33 years. The comparison of demographic and clinical variables between the two groups showed no statistically significant differences, indicating comparability in baseline characteristics. The age distribution was similar, with the majority of participants aged 41-60 years, and nearly identical mean ages (45.93 ± 15.41 in Group A vs. 46.30 ± 12.33 in Group B; p = 0.32). Evaluation of personal history revealed a higher proportion of smoking and alcohol use in Group B (81.4% and 60.5%, respectively) compared to Group A (69.8% and 53.5%, respectively), although these differences were not statistically significant. Likewise, the prevalence of comorbid conditions such as hypertension, diabetes, ischemic heart disease (IHD), chronic obstructive pulmonary disease (COPD), hypothyroidism, and benign prostatic hyperplasia (BPH) did not differ significantly between the groups (p > 0.05), thereby affirming group equivalence for outcome assessment (Table 1).

**Table 1:** Comparison of demographic and clinical profiles between the groups Group A: cases with bupivacaine-soaked mesh; Group B: controls with non-soaked polypropylene mesh

Variables		Group A (n=43), n (%)	Group B (n=43), n (%)	Chi-Square	P-value
Age Group	18-30	9 (20.9%)	6 (13.9%)	4.62	0.32
	31-40	7 (16.3%)	8 (18.6%)		
	41-50	6 (13.95%)	12 (27.9%)		
	51-60	12 (27.9%)	13 (30.2%)		
	>60	9 (20.9%)	4 (9.3%)		
	Mean ± SD	45.93 ± 15.41	46.30 ± 12.33		
Personal History	Smoking	30 (69.8%)	35 (81.4%)	1.55	0.21
	Alcohol	23 (53.5%)	26 (60.5%)	0.42	0.51
Co-morbidities	Hypertension	6 (13.9%)	7 (16.3%)	0.07	0.78
	Type 2 Diabetes	8 (18.6%)	9 (20.9%)	0.05	0.80
	Others (IHD, COPD, Hypothyroidism, BPH)	20 (47.0%)	21 (48.8%)	0.02	0.87
	None	9 (20.9%)	6 (14.0%)	0.60	0.43

Postoperative pain intensity, as assessed by the VAS, showed significantly better outcomes in Group A at all observed time intervals. At six hours postoperatively, the majority of Group A participants reported VAS scores of 3 or 4, whereas Group B exhibited higher pain levels, with 41.9% scoring 5 and 7.0% scoring 6 (χ² = 16.77, p = 0.0008). This pattern persisted at 12 hours, where no Group A patient scored above 4, in contrast to Group B, where 23.3% scored 5 and 4.7% scored 6 (χ² = 14.99, p = 0.0018). By 24 hours, all Group A participants recorded VAS scores of ≤2, while 34.9% of Group B still reported a VAS score of 3 (χ² = 27.26, p < 0.0001), indicating sustained analgesic superiority in Group A (Table 2). 

**Table 2:** Comparison of VAS scores at 6, 12, and 24 hours between the two groups Group A: cases with bupivacaine-soaked mesh; Group B: controls with non-soaked polypropylene mesh VAS: Visual Analog Scale

Variables		Group A (n=43), n (%)	Group B (n=43), n (%)	Chi-Square	P-value
Vas Score at 6 hours	3	7 (16.3%)	1 (2.3%)	16.77	0.0008
	4	31 (72.1%)	21 (48.8%)		
	5	5 (11.6%)	18 (41.9%)		
	6	0 (0.0%)	3 (7.0%)		
Vas Score at 12 hours	3	19 (44.2%)	10 (23.3%)	14.99	0.0018
	4	24 (55.8%)	21 (48.8%)		
	5	0 (0.0%)	10 (23.3%)		
	6	0 (0.0%)	2 (4.7%)		
VAS Score at 24 hours	1	15 (34.9%)	1 (2.3%)	27.26	< 0.0001
	2	28 (65.1%)	27 (62.8%)		
	3	0 (0.0%)	15 (34.9%)		

Further validation using t-test analysis confirmed statistically significant differences in mean VAS scores between the two groups at all time points: six hours (p < 0.0001), 12 hours (p = 0.0004), and 24 hours (p < 0.0001), with Group A consistently demonstrating lower pain scores, thus substantiating the enhanced analgesic efficacy of the intervention used in this group (Table 3).

**Table 3:** Comparison of VAS scores between the two groups Group A: cases with bupivacaine-soaked mesh; Group B: controls with non-soaked polypropylene mesh VAS: Visual Analog Scale

Variables	Group A (n=43), mean ± SD	Group B (n=43), mean ± SD	t-value	p-value
VAS scores at 6 hr	3.95 ± 0.53	4.53 ± 0.68	4.41	< 0.0001
VAS scores at 12 hr	3.56 ± 0.50	4.09 ± 0.8	3.68	0.0004
VAS scores at 24 hr	1.65 ± 0.48	2.32 ± 0.52	6.20	< 0.0001

With respect to additional analgesic requirements and recovery outcomes, Group A participants demonstrated significantly reduced dependence on supplemental diclofenac, with most requiring only one or two doses, whereas nearly half of Group B required three doses (Chi-square = 12.46, p = 0.0004). Additionally, Group A patients experienced a more rapid recovery, as evidenced by 67.4% being discharged by day 3 compared to only 4.7% in Group B. Conversely, most Group B patients required hospital stays of four to five days or more, highlighting a statistically significant difference in hospitalization duration (Chi-square = 47.80, p < 0.0001). These findings reinforce the clinical benefit of the analgesic strategy employed in Group A in terms of both pain control and faster postoperative recovery (Table 4). 

**Table 4:** Comparison of additional analgesic use and hospital stay duration between the two groups Group A: cases with bupivacaine-soaked mesh; Group B: controls with non-soaked polypropylene mesh

Variables		Group A (n=43), n (%)	Group B (n=43), n (%)	Chi-Square	P-value
Frequency of additional analgesics used (diclofenac)	1	11 (25.6%)	0 (0%)	12.46	0.0004
	>1	32 (74.4%)	43 (100%)		
Length of stay	3 days	29 (67.4%)	2 (4.7%)	47.80	< 0.0001
	4 days	14 (32.6%)	17 (39.5%)		
	5 days	0 (0.0%)	18 (41.9%)		
	6 days	0 (0.0%)	5 (11.6%)		
	7 days	0 (0.0%)	1 (2.3%)		

## Discussion

Age distribution in our study was broad, and although mean ages were comparable between groups, age-related factors merit discussion. Elderly patients often exhibit altered pharmacokinetics due to reduced hepatic metabolism, impaired renal clearance, and changes in body composition, which can prolong the action of local anesthetics such as bupivacaine [10,11]. In addition, comorbidities like diabetes or vascular disease may influence both wound healing and pain perception. While these variables were not independently analyzed in our study, they highlight the importance of tailoring analgesic strategies to patient characteristics, as drug handling and response may differ between younger and older individuals.

Postoperative pain, assessed by the VAS score at various intervals, showed significantly lower pain in Group A (bupivacaine-soaked mesh) compared to Group B. At six hours, Group A had a mean VAS score of 3.95 ± 0.53 vs. 4.53 ± 0.68 in Group B (p = 0.0008). At 12 hours, Group A reported 3.56 ± 0.50 vs. 4.09 ± 0.8 in Group B (p = 0.0018). At 24 hours, Group A had 1.65 ± 0.48 vs. 2.32 ± 0.52 in Group B (p = 0.0008). These results clearly indicate that patients receiving bupivacaine-soaked mesh experienced less postoperative pain.

The reduced pain perception in Group A also translated into lower requirements for additional analgesia. Only 25.6% of Group A patients required a single additional dose, while 74.4% needed it more than once. In contrast, 100% of Group B patients needed more than one dose, showing a statistically significant difference (p = 0.0004). This suggests enhanced analgesic efficacy of the bupivacaine-soaked mesh.

Chawla et al. noted that local anesthetic use at the surgeon’s discretion did not significantly alter outcomes due to randomization and blinding [7]. Similarly, Khan et al. found no significant difference in pain scores between intraperitoneal lignocaine and bupivacaine groups during laparoscopic cholecystectomy [12]. In contrast, Kamani et al. [13] demonstrated that the modified technique significantly reduced postoperative pain (VAS 0.15 vs. 0.31, p = 0.008), with a moderate effect size (Cohen’s d = 0.40), corroborated by O’Dwyer et al. [14] and Ahire et al. [15].

Romano et al. further supported these findings, showing consistently lower pain scores in patients undergoing the ProFlor technique versus the conventional Lichtenstein repair on POD 0 and 10 (VAS 2.6 vs. 5.7 and 0.2 vs. 3.1, respectively; p = 0.016) [16]. Their findings are consistent with our results, which show that bupivacaine-soaked mesh offers measurable analgesic benefit in open inguinal hernia surgery. By focusing on open repairs rather than laparoscopic or unrelated procedures, our study reinforces the direct applicability of bupivacaine as an adjunct for pain relief in standard Lichtenstein repair.

The length of hospital stay was also shorter in Group A, with 67.4% discharged within three days, compared to just 4.7% in Group B. Patients in Group B more frequently required four to seven days of hospitalization. This difference was statistically significant (p = 0.0001). Although Chawla et al. did not find significant reductions in hospital stay or analgesic use, they recommended studies with sample sizes powered for these outcomes [7].

Other studies, like that by Erhan et al., comparing mesh fixation methods (sutures, fibrin glue, cyanoacrylate) found no significant differences in operation time or hospital stay, although glue methods had fewer complications [17]. Bellows and Berger also reported slightly reduced analgesic use and hospital stay with local anesthetics, but without statistical significance [18]. Pavlin et al. found that combined rofecoxib and bupivacaine use decreased pain severity and opioid needs [19]. Callesen et al. [20] and Tverskoy et al. [21] confirmed that deeper and preemptive infiltration techniques offer superior pain relief.

Although the present study supports shorter hospital stays with bupivacaine use, the literature reveals conflicting findings. While Kamani et al. [13], Iftikhar et al. [22], and Kerawala et al. [23] found marginal benefits, large-scale reviews, including a Cochrane review of 41 studies [24], report no consistent differences. Some studies, like those by Pironi et al. [25], Neumayer et al. [26], and Mahon et al. [27], observed longer stays with certain surgical techniques. A 2009 audit reported an average stay of 3.7 days for hernia repairs, with longer durations for bilateral repairs or concurrent procedures [28].

Based on the above findings, the use of bupivacaine-soaked polypropylene mesh emerges as a superior technique for postoperative pain management in hernia repair. Bupivacaine, a long-acting amide local anesthetic, acts by blocking voltage-gated sodium channels, effectively reducing nociceptive transmission and providing sustained analgesia. When integrated with polypropylene mesh placement, this approach not only ensures effective structural reinforcement but also significantly reduces postoperative VAS scores, decreases the need for additional analgesia, shortens hospital stay, and minimizes opioid consumption. Collectively, these benefits contribute to improved patient comfort, a lower risk of chronic postoperative pain, and an overall enhanced recovery profile [19].

When considering established analgesic techniques in hernia surgery, various modalities such as local infiltration at the surgical site, ilioinguinal/iliohypogastric nerve blocks, and transversus abdominis plane (TAP) blocks have been widely studied. Callesen et al. [20] and Tverskoy et al. [21] reported superior pain relief with preemptive infiltration and nerve blocks compared to systemic analgesics alone. Our findings suggest that bupivacaine-soaked mesh offers comparable benefits, with the added advantage of providing continuous local drug release at the repair site without requiring additional injections or procedures. Thus, mesh impregnation with bupivacaine can be considered a practical, low-cost alternative or adjunct to established regional techniques for postoperative pain control in open inguinal hernia repair.

This study has several limitations. Blinding was not feasible due to the visible difference in mesh preparation, which may have introduced assessment bias. Being a single-center study with a relatively small sample size limits the generalizability of the findings. The follow-up was restricted to the immediate postoperative period, preventing evaluation of long-term outcomes such as chronic pain, recurrence, or quality of life. Moreover, the subjective nature of pain reporting and potential confounding from factors like operative time and surgical technique may have influenced results. Future multi-center studies with larger cohorts and extended follow-up are needed to validate these findings and explore comparisons with other analgesic or drug-eluting strategies.

## Conclusions

Our study demonstrated that patients in whom bupivacaine-soaked mesh was used experienced significantly less postoperative pain, with lower mean VAS scores at six, 12, and 24 hours compared to those who received non-soaked mesh. Furthermore, the need for additional analgesics was less frequent in the bupivacaine group, with fewer patients requiring more than one dose. A significantly higher proportion of patients in this group also had a shorter hospital stay of three days, highlighting the enhanced recovery profile. These findings collectively indicate that the use of bupivacaine-soaked mesh is effective in reducing postoperative pain, minimizing analgesic requirements, and promoting earlier discharge compared to conventional mesh use.
